# Burden of anemia in the United States from 1990 to 2019: a systematic analysis of the Global Burden of Disease Study 2019

**DOI:** 10.3389/fpubh.2025.1653222

**Published:** 2025-10-03

**Authors:** Michelle Shwarz

**Affiliations:** American Regent, Inc., Shirley, NY, United States

**Keywords:** anemia, DALY, Global Burden of Disease, epidemiology, GBD

## Abstract

**Background:**

Anemia is a common condition that affects both male and female individuals of all ages worldwide; however, comprehensive data on the burden of anemia and its trends over time in the United States (US) are scarce. The Global Burden of Disease Study (GBD) 2019 published global age-standardized point prevalence and disability-adjusted life years (DALYs) for 204 countries and territories between 1990 and 2019. In this study, we analyzed the GBD 2019 data to explore the factors contributing to the burden of anemia in the US.

**Methods:**

In this secondary analysis of the GBD 2019 data, we extracted point estimates and 95% uncertainty intervals of DALYs to describe the health loss due to anemia in the US between 1990 and 2019. We categorized DALYs based on demographic characteristics and attributed them to anemia-causing conditions, such as dietary iron deficiency (DID), diabetes, kidney diseases, digestive diseases, and obstetric or gynecological conditions.

**Findings:**

From 1990 to 2019, anemia-related DALYs increased from 332,449 to 418,855 in the US; more than half of the DALYs were due to DID. The rates of DALYs per 100,000 people declined steadily from 1990 until the early 2000s, after which it began to increase steadily. Notably, the rates of DALYs were higher in female individuals than in male individuals for anemia due to DID, diabetes and kidney diseases, and digestive diseases up to the age of 80. There were significant variations in the health burden of anemia across different states, with the highest rates observed in Mississippi, the District of Columbia, and Alabama.

**Conclusion:**

These data on the burden of anemia—measured in years of health lost—and its upward trend over time in the US highlight the need for screening, diagnosis, and treatment of anemia to guarantee health access for all.

## Introduction

The World Health Organization (WHO) defines anemia as “a condition in which the number of red blood cells or the hemoglobin concentration within them is lower than normal” (1). The resulting decrease in oxygen delivery to tissues has widespread impacts on the body, with different effects on pregnant women, children, and adults (2). Anemia is also associated with an increased risk of all-cause mortality and hospitalizations among adults aged ≥66 years who have cognitive decline and an elevated incidence of dementia in adults aged ≥65 years (3, 4). Pregnant women are at a higher risk of anemia due to the increased maternal blood volume and the fetus’s demand for iron (5).

The global prevalence of anemia in 2021 was 24.3% (95% uncertainty interval [UI]: 23.9–24.7), equating to 1.92 billion cases (95% UI, 1.89–1.95), with sex, age, and geography being associated with differences in the burden (2). The prevalence of anemia in the United States (US) grew from 6.5% (6.2–6.9) in 1990 to 7.0% (6.4–7.7) in 2021 (2). Recent studies based on the National Health and Nutrition Examination Survey (NHANES) data in the US have documented increases in the prevalence of anemia, showing varying impacts based on age, ethnicity, and sex (6–9). The US Department of Health and Human Services has developed the Healthy People 2030 Framework to support key public health goals in the country, with two objectives aimed at reducing iron deficiency: one focused on children (NWS-16) and the other on female individuals (NWS-17) (10, 11).

Globally and in the US, dietary iron deficiency (DID) is the most common cause of anemia (2). Anemia of inflammation is considered to be the second most prevalent cause of anemia worldwide. It is particularly common among patients with chronic diseases and those who are hospitalized (12). In the US, conditions other than DID that can cause anemia include chronic kidney disease (CKD); hemoglobinopathies and hemolytic anemias; infectious diseases; neglected tropical diseases; endocrine, metabolic, blood, and immune disorders; upper digestive system diseases; inflammatory bowel disease; gynecological diseases and maternal disorders (in female individuals); HIV/AIDS; vitamin A deficiency; cirrhosis and other chronic liver diseases; and parasitic infections (such as malaria, schistosomiasis, and nematode infections) (2).

The Global Burden of Disease Study (GBD) is a comprehensive and systematic effort that quantifies the burden of all major diseases, risk factors, and outcomes, using standardized summary metrics, such as disability-adjusted life years (DALYs); unlike prevalence measures, DALYs allow for standardized comparisons over time, across populations, and between diseases (13). As anemia is a multifactorial disease with diverse effects, DALYs are a useful tool for measuring the burden of anemia (14). Subnational GBD assessments of disease burden in India, Ethiopia, and Brazil have been published, highlighting the need for fine-grained data that aid evidence-based decision-making in diverse societies (13). In this study, we conducted a secondary analysis of the GBD 2019 data at the subnational level to understand the major factors contributing to the burden of anemia in the US, the influence of demographic characteristics, and the extent of variation among the states in the US. The objective of this study was to use the GBD 2019 data to quantify the burden of anemia in the US from 1990 to 2019, stratified by sex, age, cause, and geography.

## Methods

The GBD study provides annual estimates of disease burden for 204 countries and territories, covering 369 diseases and 87 risk factors from 1990 to 2019 (15, 16).

The approach employed by the GBD modelers has been described in previous studies (15, 16). Data are cleaned for errors, such as miscoding and missingness, and cause-specific incidences are estimated using Bayesian meta-regression to reconcile incidence, prevalence, remission, and cause-specific mortality. Burden metrics include years of life lost (YLLs) and years lived with disability (YLDs) (17). Anemia is considered an impairment that does not directly cause mortality (18); therefore, in this analysis, DALYs are equivalent to YLDs. One DALY represents the loss of 1 year of full health (15).

### Case definitions

In the GBD 2019, anemia is defined as a reduction in the concentration of hemoglobin in the blood, irrespective of the underlying cause or the morphology or function of erythrocytes (15). In the GBD framework, anemia is classified as an impairment—a physical state with multiple causes, such as DID, diabetes and kidney diseases, digestive diseases, maternal hemorrhage, and others (15).

### Data analysis

Total anemia is estimated based on the modeled mean and standard deviation of hemoglobin concentration as inputs. Individual-level data sources are incorporated to produce an estimated hemoglobin distribution for each age-sex-location-year combination. Hemoglobin thresholds for anemia are based on the WHO standards and vary by sex, age, and pregnancy status (15, 19).

To attribute the estimated total anemia to different anemia-causing conditions, the GBD modelers estimate counterfactual hemoglobin distributions (i.e., hypothetical distributions of hemoglobin if no conditions caused anemia) based on (i) the age- and sex-specific prevalence of each anemia-causing condition and (ii) the condition’s quantitative effect on hemoglobin concentration, known as the “hemoglobin shift” for that condition (15). The total hemoglobin shift in a population is the difference between the predicted hemoglobin concentration and the “normal” hemoglobin concentration for each age-sex-location-year combination. “Normal” hemoglobin is defined as the 95th percentile of the global distribution of mean hemoglobin levels within each age group, sex, and year (14). Cause-specific hemoglobin shifts are derived by meta-analysis of published studies (cohort studies, case control studies, or treatment trials) that compare hemoglobin concentrations among populations with and without the condition and are described in detail elsewhere (14). Anemia severity levels (mild, moderate, and severe) are assigned to each cause based on the difference between the counterfactual and observed hemoglobin distributions in each population group, forcing the sum of the difference between the counterfactual and observed prevalence of anemia across causes to equal the total anemia of each severity level.

Point estimates and 95% UIs generated by the GBD 2019 modelers are reported. The uncertainty ranges in the estimated DALYs incorporate the uncertainty in the prevalence and the uncertainty resulting from the modeling process to generate the disability weight (15). A global standard reference population, developed by demographers at the Institute for Health Metrics and Evaluation, is used to derive an age-standardized rate to provide a weighted average of age-specific rates for each population. This allows comparisons over time, independent of the age structure of each population.

DALYs from the GBD 2019 dataset were analyzed by age, sex, geography, and cause to assess anemia’s health impact in the US. This study focused on dietary iron deficiency (DID), diabetes, kidney disease, digestive disease, and maternal and gynecological conditions.

### Role of the funding source

The funder had no role in the primary data collection or study design of the GBD study. However, the funder was involved in the study design, data analysis, and provided support for the medical writing of the secondary analysis described here.

## Results

### Dietary iron deficiency as a significant cause of the anemia burden in the US

In 2019, an estimated 218,855 (95% UI, 257,649–657,344) DALYs were lost to anemia in the US, with over half attributed to dietary iron deficiency (DID) (228,240 [140,291–360,026]). DID accounted for the largest share of DALYs in both male and female individuals (Table 1), with 61,499 DALYs among male individuals and 166,741 among female individuals—nearly three times more.

**Table 1:** Burden of anemia in the US in 2019 by sex among all ages and individuals aged 10–54 years.

Anemia cause by age	DALYs (95% UI) in both sexes	DALY rates (95% UI) per 100,000 in both sexes	DALYs (95% UI) in male individuals	DALYs (95% UI) in female individuals	DALY rates (95% UI) per 100,000 male individuals	DALY rates (95% UI) per 100,000 female individuals
All ages						
Anemia	418,855 (257,649-657,344)	127.7 (78.6–200.4)	108,182 (61,422–180,634)	310,673 (190,806–483,052)	67.1 (38.1–112.0)	186.4 (114.5–289.8)
DID	228,240 (140,291-360,026)	69.6 (42.8–109.8)	61,499 (33,956–104,047)	166,741 (101,198–264,114)	38.1 (21.1–64.5)	100.0 (60.7–158.4)
Diabetes and kidney diseases	65,888 (40,058-102,376)	20.1 (12.2–31.2)	23,006 (12,811–36,145)	42,882 (25,540–69,131)	14.3 (7.9–22.4)	25.7 (15.3–41.5)
Digestive diseases	12,195 (7,550–18,644)	3.7 (2.3–5.7)	1,861 (1,068–3,030)	10,334 (6,361–15,888)	1.2 (0.7–1.9)	6.2 (3.8–9.5)
Gynecological diseases	10,568 (5,908–18,043)	3.2 (1.8–5.5)	NA	10,568 (5,908–18,043)	NA	6.3 (3.5–10.8)
Maternal hemorrhage	3,660 (2,024–6,121)	1.1 (0.6–1.9)	NA	3,660 (2,024–6,121)	NA	2.2 (1.2–3.7)
Aged 10–54 years						
Anemia	217,105 (127,751–343, 586)	112.6 (66.3–178.2)	28,365 (14,424–52,125)	188,741 (109,805–301,869)	29.3 (14.9–53.8)	196.7 (114.4–314.6)
DID	127,368 (73,706–203,772)	66.1 (38.2–105.7)	20,344 (10,313–37,622)	107,024 (61,074–173,476)	21.0 (10.7–38.9)	111.5 (63.7–180.8)
Diabetes and kidney diseases	7,230 (4,195–11,675)	3.8 (2.2–6.1)	781 (383–1,439)	6,449 (3,743–10,411)	0.8 (0.4–1.5)	6.7 (3.9–10.9)
Digestive diseases	7,166 (4,313–11,147)	3.7 (2.2–5.8)	315 (152–597)	6,851 (4,137–10,706)	0.3 (0.2–0.6)	7.1 (4.3–11.2)
Gynecological diseases	10,166 (5,605–17,313)	5.3 (2.9–9.0)	NA	10,166 (5,605–17,313)	NA	10.6 (5.8–18.0)
Maternal hemorrhage	3,660 (2,024–6,121)	1.9 (1.0–3.2)	NA	3,660 (2,024–6,121)	NA	3.8 (2.1–6.4)

DALY, disability-adjusted life year; DID, dietary iron deficiency; NA, not applicable; UI, uncertainty interval; US, United States.

Over half of the overall anemia-related DALYs were experienced by those in the 10–54 age group (51.8%; 217,105 [127,751–343,586]; Table 1). Similar to the overall trend, a large share of the anemia-related DALYs were due to DID (127,368 [73,706–203,772]; Table 1), with female individuals in this age group experiencing higher rates of DALYs than male individuals (Table 1).

### Other significant causes of anemia burden

Two major cause categories—diabetes and kidney diseases and digestive diseases—together contributed a significant portion of anemia-related DALYs (65,888 and 12,195, respectively), making them the second-largest causes of the anemia burden. A smaller proportion of anemia-related DALYs among female individuals was due to maternal hemorrhage (3,660 [2,024–6,121]) and gynecological diseases (10,568 [5,908–18,043]) (Table 1). As expected, this burden was largely experienced by female individuals in the 10–54 age group, who accounted for all of the burden of maternal hemorrhage (100%; 3,660 [2,024–6,121]) and most of the burden of gynecological diseases (96.2%; 10,166 [5,605–17,313]; Table 1). Anemia-related DALYs caused by gynecological diseases in this age group were divided into two categories: DALYs due to uterine fibroids (1,118 [666–1,779]) and those due to other gynecological diseases (9,048 [4,749–15,740]).

### Differences in anemia burden by sex

Female individuals accounted for 74.2% (310,673) of the total anemia-related DALYs, with 188,741 of these in female individuals aged 10–54 years. Across all age groups, female individuals experienced higher anemia-related DALY rates than male individuals (Table 1; Supplementary Table 2).

Among female individuals in each age group, DID was the foremost cause of anemia-related DALYs (Supplementary Table 1). Among female individuals aged 0–9 years and 10–54 years, hemoglobinopathies and hemolytic anemias were the second leading cause of anemia-related DALYs. In contrast, among female individuals aged ≥55 years, diabetes and kidney diseases were the second leading cause of anemia-related DALYs (Supplementary Table 1). Although male and female individuals aged ≥55 years experienced generally high rates of anemia-related DALYs due to DID, digestive diseases, and diabetes and kidney diseases, DALY rates per 100,000 female individuals were comparatively higher than those for 100,000 male individuals for each cause (Supplementary Table 2).

**Figure 1:**
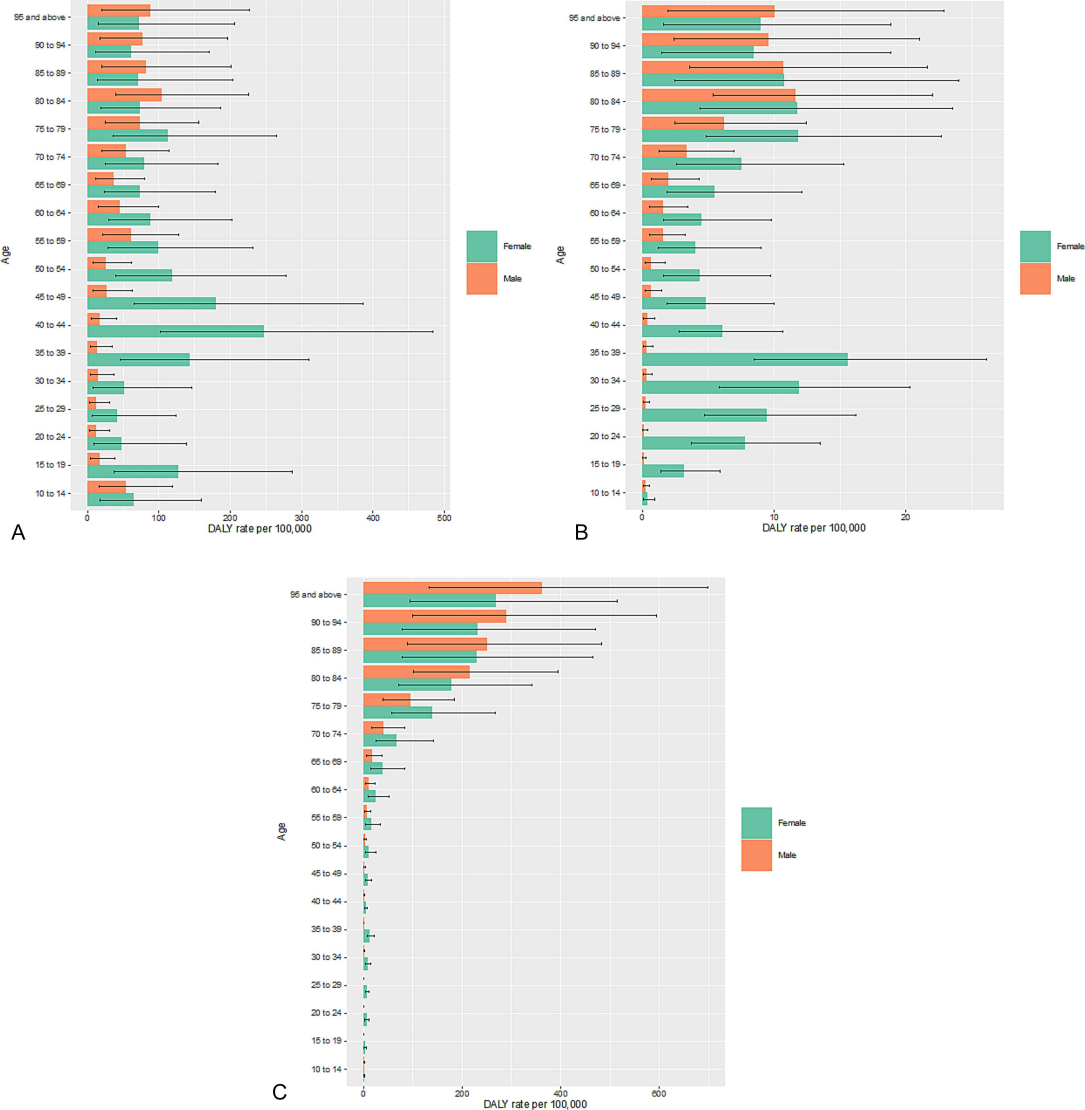
Rates of anemia-related DALYs per 100,000 due to **(A)** DID, **(B)** digestive diseases, and **(C)** diabetes and kidney diseases in both sexes in the US in 2019. DALY, disability-adjusted life year; DID, dietary iron deficiency; US, United States.

An in-depth exploration of the DALY rates among different age groups revealed starkly different trends between male and female individuals. Female individuals aged 35–44 years experienced the highest anemia-related DALY rates due to DID and digestive diseases, whereas the corresponding rates for male individuals increased steadily with age. However, male individuals had higher DALY rates than female individuals in the ≥80-year age group for DID and in the ≥90-year age group for digestive diseases (Figures 1A,B). DALY rates for anemia due to diabetes and kidney diseases were generally higher among female individuals than male individuals in age groups <80 years and increased somewhat steadily with age rather than spiking in female individuals in their 30s and 40s, as seen in DID and digestive diseases (Figure 1C).

### Burden of severe anemia

The burden of severe anemia followed a trend similar to that of overall anemia. The DALY rate among female individuals of all ages was three times that of male individuals of all ages (Table 2). Among those aged 10–54 years, female individuals had a DALY rate approximately 17 times higher than that of male individuals for severe anemia (Table 2). Similarly, female individuals of all ages had a DALY rate for severe anemia due to DID approximately three times that of male individuals, and female individuals aged 10–54 years had a DALY rate for severe anemia due to DID approximately 10 times that of male individuals (Table 2). Diabetes and kidney disease were also major causes of severe anemia in both sexes (Table 2).

**Table 2:** Burden of severe anemia in the US in 2019 by sex among all ages and individuals aged 10–54 years.

Anemia cause by age	DALYs (95% UI) in both sexes	DALY rates (95% UI) per 100,000 in both sexes	DALYs (95% UI) in male individuals	DALYs (95% UI) in female individuals	DALY rates (95% UI) per 100,000 male individuals	DALY rates (95% UI) per 100,000 female individuals
All ages						
Severe anemia	34,974 (22,389–52,054)	11 (7–16)	8,631 (5,372–12,850)	26,343 (16,318–41,213)	5 (3–8)	16 (10–25)
DID	19,225 (12,041–29,396)	6 (4–9)	4,418 (2,674–6,656)	14,807 (8,945–23,771)	3 (2–4)	9 (5–14)
Diabetes and kidney diseases	6,097 (3,891–8,924)	2 (1–3)	2,520 (1,511–3,910)	3,577 (2,129–5,666)	2 (1–2)	2 (1–3)
Digestive diseases	951 (610–1,400)	0.3 (0.2–0.4)	177 (108–271)	774 (484–1,190)	0.1 (0.1–0.2)	0.5 (0.3–0.7)
Gynecological diseases	750 (398–1,369)	0.2 (0.1–0.4)	NA	750 (398–1,369)	NA	0.4 (0.2–0.8)
Maternal hemorrhage	258 (134–458)	0.1 (0–0.1)	NA	258 (134–458)	NA	0.2 (0.1–0.3)
Ages 10–54 years						
Severe anemia	17,538 (9,987–29,572)	9 (5–15)	1,443 (732–2,522)	16,096 (8,839–27,549)	1 (1–3)	17 (9–29)
DID	10,769 (6,010–18,344)	6 (3–10)	1,054 (532–1,879)	9,715 (5,202–17,036)	1 (1–2)	10 (5–18)
Diabetes and kidney diseases	506 (299–851)	0.3 (0.2–0.4)	34 (18–60)	472 (274–810)	<0.1	0.5 (0.3–0.8)
Digestive diseases	510 (299–816)	0.3 (0.2–0.4)	13 (7–22)	497 (287–805)	<0.1	0.5 (0.3–0.8)
Gynecological diseases	721 (374–1,338)	0.4 (0.2–0.7)	NA	721 (374–1,338)	NA	0.8 (0.4–1)
Maternal hemorrhage	258 (134–458)	0.1 (0.1–0.2)	NA	258 (134–458)	NA	0.3 (0.1–0.5)

DALY, disability-adjusted life year; DID, dietary iron deficiency; NA, not applicable; UI, uncertainty interval; US, United States.

### Historic trends in anemia burden

**Figure 2:**
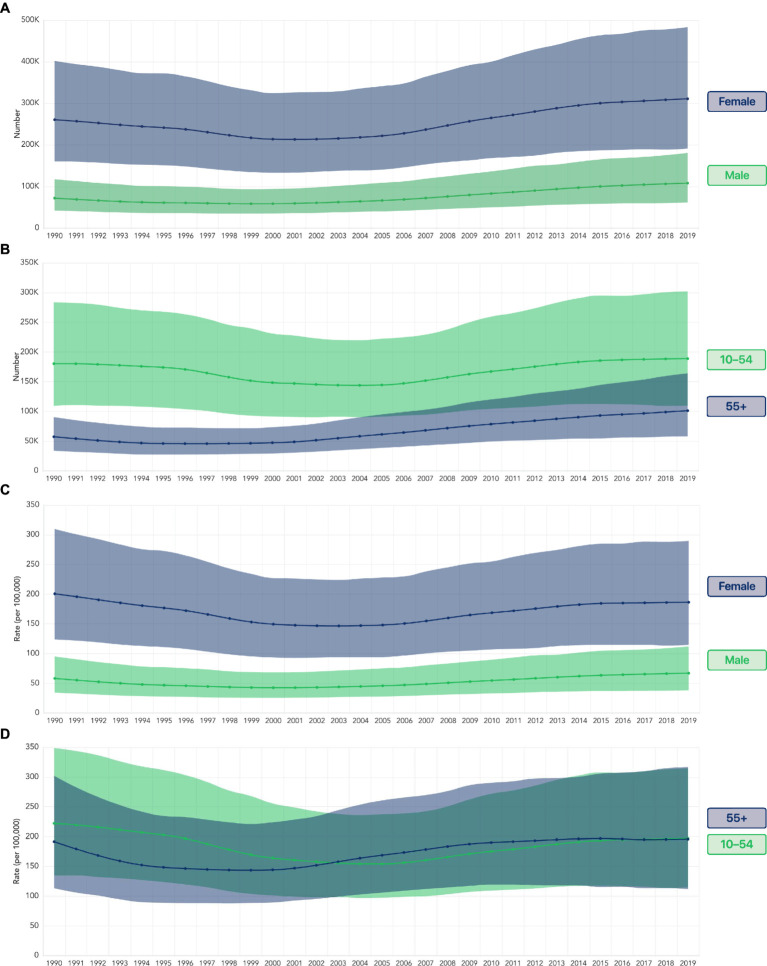
**(A)** Number of anemia-related DALYs in the US by sex. **(B)** Number of anemia-related DALYs in the US by age group in female individuals. **(C)** Rate of anemia-related DALYs per 100,000 in the US by sex. **(D)** Rate of anemia-related DALYs per 100,000 female individuals in the US by age group. Shading indicates uncertainty intervals. DALY, disability-adjusted life year.

From 1990 to 2019, total health loss due to anemia in the US rose from 332,449 (204,344–510,889) to 418,855 (257,649–657,344), representing an increase of 26% over three decades. In female individuals, this increase was most prominent since 2001 (Figure 2A). Female individuals experienced 212,930 (133,559–326,136) anemia-related DALYs in 2001, which then rose by 46% to 310,673 (190,806–483,052) in 2019. Among female individuals aged ≥55 years, there was an even sharper increase, more than doubling from 48,775 (30,687–75,803) in 2001 to 101,293 (57,831–164,094) in 2019 (Figure 2B).

The DALY rates per 100,000 female individuals indicate that some of this increase in health loss may be due to demographic changes, such as an increase in the number of female individuals in the ≥55 years group. In 2019, female individuals experienced lower DALY rates than in 1990, although rates have been increasing since the early 2000s. The lowest estimated DALY rate among female individuals in the US occurred in 2003, at 146 (94–224) DALYs per 100,000 female individuals (Figure 2C). Although DALY rates among male individuals were far lower than those in female individuals, their trend was similar, decreasing from 58 (34–95) in 1990 until the early 2000s, then increasing to reach 67 (38–112) in 2019.

DALY rates in 1990 were different between female individuals aged 10–54 years and those aged ≥55 years. However, DALY rates have since risen from their respective low points in the 2000s for each group and increased in 2019 to 196 (112–317) for female individuals aged 10–54 years and 197 (114–315) for female individuals aged ≥55 years (Figure 2D).

### Variation in the burden of anemia by location

**Figure 3:**
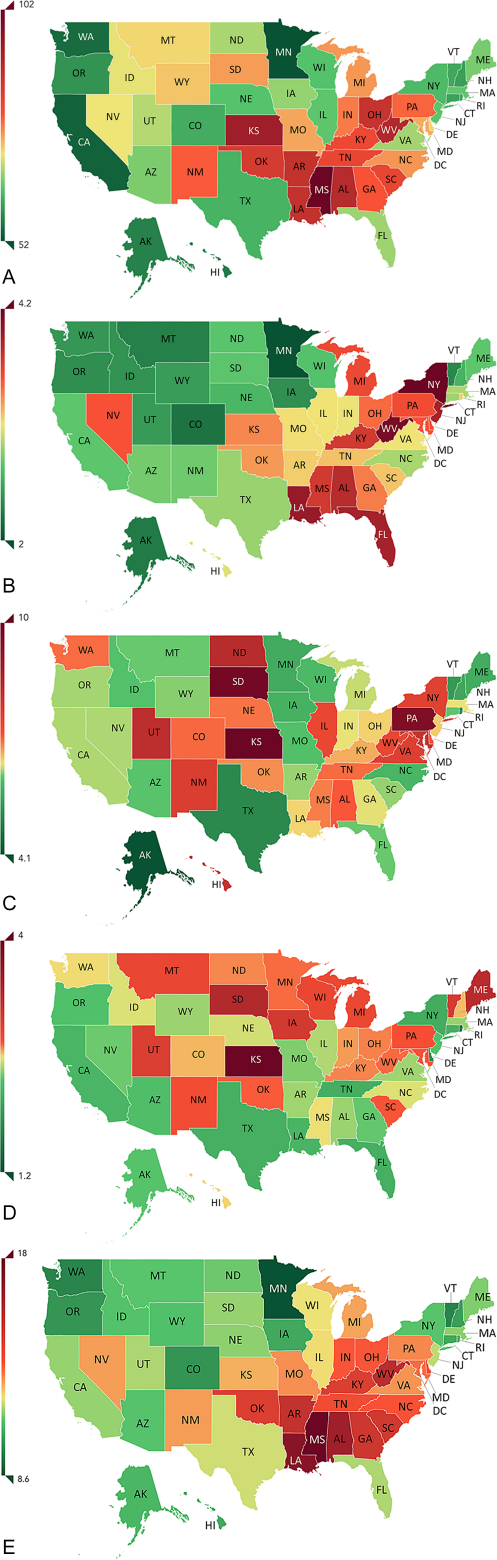
Rates of age-standardized anemia-related DALYs per 100,000 due to **(A)** dietary iron deficiency in both sexes, **(B)** digestive diseases in both sexes, **(C)** gynecological diseases in female individuals, **(D)** maternal hemorrhage in female individuals, and **(E)** diabetes and kidney diseases in both sexes, all in the US in 2019. AK, Alaska; AL, Alabama; AR, Arkansas; AZ, Arizona; CA, California; CO, Colorado; CT, Connecticut; DALY, disability-adjusted life year; DC, District of Columbia; DE, Delaware; FL, Florida; GA, Georgia; HI, Hawaii; IA, Iowa; ID, Idaho; IL, Illinois; IN, Indiana; KS, Kansas; KY, Kentucky; LA, Louisiana; MA, Massachusetts; MD, Maryland; ME, Maine; MI, Michigan; MN, Minnesota; MO, Missouri; MS, Mississippi; MT, Montana; NC, North Carolina; ND, North Dakota; NE, Nebraska; NH, New Hampshire; NJ, New Jersey; NM, New Mexico; NV, Nevada; NY, New York; OH, Ohio; OK, Oklahoma; OR, Oregon; PA, Pennsylvania; RI, Rhode Island; SC, South Carolina; SD, South Dakota; TN, Tennessee; TX, Texas; US, United States; UT, Utah; VA, Virginia; VT, Vermont; WA, Washington; WI, Wisconsin; WV, West Virginia; WY, Wyoming. United States map used with permission from Institute for Health Metrics and Evaluation (IHME).

The rates of health loss due to anemia, both overall rates and those attributed to different causes, varied among US states even after adjusting for different population sizes and age structures (Supplementary Table 3; Figures 3A–E). Age-standardized DALYs per 100,000 population in the US in 2019 ranged from 175 (102–282) in Mississippi to 91 (50–158) in Minnesota (Supplementary Table 4). Among female individuals, these rates ranged from 256 (142–423) in Mississippi to 133 (71–228) in Minnesota. Comparing 1990 and 2019, although the order of states with the highest rates of anemia-related DALYs per 100,000 changed, the states in the top 10 largely remained the same (Supplementary Table 3). Between 1990 and 2019, 16 states experienced an increase in DALY rates (Supplementary Table 4). Among the states with the top ten DALY rates in 1990, the District of Columbia, Louisiana, South Carolina, Georgia, and Maryland had lower DALY rates by 2019 (Supplementary Tables 3, 4).

DALY rates due to different causes varied widely among states. Mississippi recorded the highest rate of anemia due to DID (Figure 3A); New York and West Virginia had the highest rates of anemia due to digestive diseases (Figure 3B); South Dakota, Kansas, and Pennsylvania had the highest rates of anemia due to gynecological diseases (Figure 3C); Kansas had the highest rate of anemia due to maternal hemorrhage (Figure 3D); and Mississippi had the highest rate of anemia due to diabetes and kidney diseases (Figure 3E).

## Discussion

This study demonstrates that anemia-related DALYs in the US increased by 26% from 1990 to 2019, with female individuals accounting for nearly three-quarters of the burden. More than half of the DALYs were attributable to dietary iron deficiency, particularly among female individuals of reproductive age. These findings highlight both the growing burden of anemia and striking demographic and geographic disparities. A recent GBD study showed that in 2021, the global prevalence of anemia was 17.5% (17.0–18.0) among male individuals and 31.2% (30.7–31.7) among female individuals (2). The prevalence of anemia was >40% among children aged <2 years and the older adults aged ≥90 years (2). Geographical disparities in the prevalence were noted, with certain regions of the world (Western and Central Sub-Saharan Africa and South Asia) experiencing greater total anemia prevalence than other regions of the world (2). In the above-mentioned study, the prevalence of anemia in high-income North America increased from 6.5% (6.1–7.0) in 1990 to 6.8% (6.2–7.6) in 2021 (2). While Canada and Greenland saw decreases in anemia prevalence—from 6.5% (4.3–10.3) to 5.1% (4.0–8.0) and 8.9% (7.2–11.4) to 6.3% (5.7–7.0), respectively—anemia prevalence in the US rose from 6.5% (6.2–6.9) in 1990 to 7.0% (6.4–7.7) in 2021. This increase was not simply due to demographic changes, but there was also a corresponding increase in the rate of YLDs per 100,000 people, from 91.0 (58.6–138.9) in 1990 to 108.4 (69.4–166.5) in 2021 (2).

This study revealed that over 400,000 years were lost to disability due to anemia in the US in 2019, with three-quarters of the burden experienced by female individuals, primarily those of reproductive age, due to DID. Inflammatory conditions were the second-largest contributor to the anemia burden, consistent with previous findings showing a 50% higher risk of anemia in non-pregnant women with inflammation (20).

The DALY rates per 100,000 decreased to a low point in the early 2000s, following which both the prevalence of anemia (previously reported) (7, 8) and its associated DALYs (current study) began to rise. The difference in total anemia-related DALYs between female individuals aged 10–54 years and those aged 55 years and above may be due to a demographic shift, i.e., an increase in the number of female individuals in the ≥55 years age group, as DALY rates in 2019 have converged for these two age groups. In addition, this aging population is likely living longer with inflammatory conditions such as diabetes and kidney disease, contributing to higher overall DALYs due to anemia in this age group. This demographic shift is also reflected in the increase in DALYs due to gynecological diseases among female individuals aged ≥55 years, despite the DALY rate remaining low over the last two decades (DALY rate 0.6 [0.3–0.9] in 2001 and 0.9 [0.4–1.4] in 2019; DALYs 186 in 2001 and 402 in 2019).

The rise in anemia due to DID from 1999 to 2018 parallels a decline in dietary iron intake, with the highest DALY rates in female individuals aged 35–49 years likely due to menstruation-related iron loss (21).

The burden of anemia due to diabetes and kidney diseases in the US increased by 43% from 1990 (14 [9–22] per 100,000) to 2019 (20 [12–31] per 100,000). The same time period saw a 53.4% increase in the age-standardized prevalence of type 2 diabetes in the US (22). The age-standardized prevalence of CKD in the US increased only by 0.1% from 1990 to 2017 (23). Diabetes often leads to CKD; therefore, an increase in the prevalence of type 2 diabetes may subsequently increase the prevalence of CKD and associated anemia; however, mechanisms independent of renal impairment have also been suggested for the development of anemia in individuals with diabetes (24, 25). In addition, the prevalence of diabetes and CKD increases with age, which explains the increased health loss due to anemia associated with diabetes and kidney diseases among the older adults in our study (26, 27).

Female individuals aged 35–39 years experienced the highest anemia burden from digestive diseases, compared to male individuals up to the age of 80. Conditions such as inflammatory bowel disease, gallbladder disease, and gastritis likely contributed to these outcomes (28–30).

The rate of health loss due to anemia varied widely across the US states, both for overall anemia and anemia due to different underlying causes. For example, Hawaii had higher rates of anemia-related DALYs due to gynecological diseases and maternal conditions compared to other states, but it fared well with respect to other causes of anemia. The largest anemia burden in Michigan was due to digestive diseases, but it had lower DALY rates for gynecological diseases compared to other states. These geographic differences may be driven by variations in socioeconomic status, healthcare access, and prevalence of chronic conditions such as diabetes and kidney disease, and regional dietary patterns. States with higher rates of poverty and food insecurity, as well as limited access to preventive healthcare services, may experience a disproportionately higher anemia burden (31, 32). These factors may be compounded by the rising consumption of ultra-processed foods, which are typically lower in micronutrients, contributing to reduced dietary iron intake and increased anemia prevalence (33). This finding highlights the scope for improvement in overall anemia levels in certain geographic regions and the need for tailored policy and treatment options to address health loss due to the different underlying causes of anemia. Current guidelines highlight the different needs and treatment options available for the underlying causes of anemia (Supplementary Table 5). Anemia is a treatable condition when appropriate screening, diagnosis, and treatment options are available to patients. However, addressing anemia requires not only individual-level nutritional interventions but also cross-disciplinary approaches that improve food security, dietary quality, and nutrition education. National and state-level programs that increase access to iron-rich foods, reduce reliance on ultra-processed foods, and enhance nutrition education programming could complement clinical interventions (33–35). When prevention efforts are insufficient, timely diagnosis and appropriate clinical management, including oral and, when indicated, parenteral iron therapy, can help reduce both the burden of the disease and the cost of care (37–43).

This study has several strengths. It is the first to use the GBD 2019 data to provide a subnational, sex- and age-specific analysis of the anemia burden in the United States. The use of DALYs allows for standardized comparisons across time, demographics, and geography. These granular data provide policy-relevant insights that can guide targeted interventions at both national and state levels.

There are also limitations to this study. As each case of anemia is attributed to a specific cause, the GBD estimation does not allow for anemia to be attributed to multiple simultaneous causes (36). Not every cause of anemia is currently included in the GBD estimates; for example, there could be acute and chronic hemorrhagic states where inadequate iron intake is not the only underlying issue (15). Finally, our study focused on DID, anemia of inflammation, and gynecological and maternal disorders; however, other causes of anemia were not included as DID and anemia of inflammation are relatively more common causes of anemia in the US.

In summary, the burden of anemia is growing in the US, with DID contributing the most to health loss and female individuals experiencing the majority of the overall burden. This analysis of the GBD 2019 data highlights the burden of anemia and the need for population-based interventions to better screen, diagnose, and treat anemia, as well as to strengthen healthcare delivery systems to provide effective and cost-efficient care. The global target of a 50% reduction in anemia among female individuals of reproductive age merits replication in the US, extending to a broader population.

## Research in context

### Evidence before this study

We searched Ovid MEDLINE^®^ from 1946 to 10 January 2025 using the search terms (anemia) AND ([DALY] OR [disability-adjusted life year] OR [Global Burden of Disease]) AND (United States), with no language restrictions. This search did not yield any references. However, when the term “United States” was excluded from the search string, 24 references were retrieved. Of these references, 14 were discarded as irrelevant because they pertained to anemia in the context of parasitic infections, involved studies based on the Global Burden of Disease Study (GBD) data that were not related to anemia, or examined the cost-effectiveness of health interventions. Three studies have described the global burden of anemia based on the GBD data, along with a commentary on one of these articles and a study focused on anemia in female individuals of reproductive age in low- and middle-income countries. To the best of our knowledge, this is the first study to examine the burden of anemia in the US, segmenting the data by various demographic factors.

### Added value of this study

In this analysis, the GBD 2019 dataset was used to examine the burden of anemia in the US from 1990 to 2019 by sex, cause, age, and geographic area. To the best of our knowledge, this is the first subnational assessment of anemia in the US that examines disability-adjusted life years (DALYs) across different demographics and geographies. This study is also the first to describe the burden of anemia by age, sex, and cause, to explore geographic variation in anemia-related DALYs by cause; and to analyze historical trends of anemia-related DALYs by geography.

### Implications of all the available evidence

This analysis of the GBD 2019 data showed an increase in the burden of anemia over time. This burden was concentrated in specific groups of people (female individuals and the older adults), with some locations (Mississippi, the District of Columbia, and Alabama) experiencing a greater burden than others. These findings highlight the importance of continued efforts toward the screening, diagnosis, and management of anemia. The World Health Organization has declared anemia a global health priority, with anemia testing included in the essential list of *in vitro* diagnostics. The comprehensive nature of our study provides policy-relevant information to guide public health programs in addressing anemia care for specific populations in the US.
