# Supplementary material for: Burden of anemia in the United States from 1990 to 2019: a systematic analysis of the Global Burden of Disease Study 2019
Source: Front Public Health. 2025 Oct 3;13:1653222. doi: 10.3389/fpubh.2025.1653222 (PMC12532043; doi:10.3389/fpubh.2025.1653222)
Supplement: Supplementary file 1 [file Table_1.DOCX]

**Supplementary Table 1. Causes of anemia burden associated with >1000 DALYs (95% UI) per year among females in the US in 2019.**

| **Cause** | **DALYs (95% UI)** |
| --- | --- |
| **Females** | 310,673 (190,806–483,052) |
| **Aged 0–9 years** | 20,639 (7990–41,678) |
| Dietary iron deficiency | 15,092 (5816–30,639) |
| Hemoglobinopathies and hemolytic anemias | 2148 (844–4287) |
| Neglected tropical diseases and malaria | 1462 (556–2992) |
| **Aged 10–54 years** | 188,741 (109,805–301,869) |
| Dietary iron deficiency | 107,024 (61,074–173,476) |
| Hemoglobinopathies and hemolytic anemias | 32,268 (18,789–51,997) |
| Endocrine, metabolic, blood, and immune disorders | 10,239 (5768–16,370) |
| Gynecological diseases | 10,166 (5605–17,313) |
| Uterine fibroids | 1118 (666–1779) |
| Other gynecological diseases | 9048 (4749–15,740) |
| Digestive diseases | 6851 (4137–10,706) |
| Diabetes and kidney diseases | 6449 (3743–10,411) |
| Neglected tropical diseases and malaria | 5067 (2890–8199) |
| Other infectious diseases | 4837 (2762–7973) |
| Maternal hemorrhage | 3660 (2024–6121) |
| HIV/AIDS and sexually transmitted infections | 2138 (1024–3812) |
| **Aged ≥55 years** | 101,293 (57,831–164,094) |
| Dietary iron deficiency | 44,625 (24,368–75,735) |
| Diabetes and kidney diseases | 36,328 (21,121–59,272) |
| Hemoglobinopathies and hemolytic anemias | 10,121 (5774–16,591) |
| Endocrine, metabolic, blood, and immune disorders | 3544 (1877–6062) |
| Digestive diseases | 3446 (1953–5603) |
| Other infectious diseases | 1305 (681–2285) |
| Neglected tropical diseases and malaria | 1304 (684–2351) |

DALY, disability-adjusted life year; UI, uncertainty interval; US, United States.
