# Supplementary material for: Burden of anemia in the United States from 1990 to 2019: a systematic analysis of the Global Burden of Disease Study 2019
Source: Front Public Health. 2025 Oct 3;13:1653222. doi: 10.3389/fpubh.2025.1653222 (PMC12532043; doi:10.3389/fpubh.2025.1653222)
Supplement: Supplementary file 2 [file Table_2.DOCX]

**Supplementary Table 2. Cause-specific anemia DALY rates among males and females aged ≥55 years in the US in 2019.**

| **Cause** | **DALY rates (95% UI) per 100,000 males** | **DALY rates (95% UI) per 100,000 females** |
| --- | --- | --- |
| DID | 56.2 (29.5–97.2) | 86.2 (47.0–146.2) |
| Digestive diseases | 3.4 (1.9–5.5) | 6.7 (3.8–10.8) |
| Diabetes and kidney diseases | 49.2 (27.4–78.0) | 70.1 (40.8–114.4) |

DALY, disability-adjusted life
