# Supplementary material for: Burden of anemia in the United States from 1990 to 2019: a systematic analysis of the Global Burden of Disease Study 2019
Source: Front Public Health. 2025 Oct 3;13:1653222. doi: 10.3389/fpubh.2025.1653222 (PMC12532043; doi:10.3389/fpubh.2025.1653222)
Supplement: Supplementary file 3 [file Table_3.DOCX]

**Supplementary Table 3. Top 10 US states in order of highest age-standardized rates of DALYs per 100,000 due to anemia in both sexes in 1990 and 2019.**

| **1990** | | **2019** | |
| --- | --- | --- | --- |
| **State** | **DALY rates (95% UI)** | **State** | **DALY rates (95% UI)** |
| District of Columbia | 260 (158–408) | Mississippi | 175 (102–282) |
| Mississippi | 170 (97–277) | District of Columbia | 166 (98–268) |
| Louisiana | 162 (94–269) | Alabama | 158 (92–252) |
| South Carolina | 159 (89–259) | Kansas | 157 (91–258) |
| Alabama | 157 (90–254) | Louisiana | 155 (89–250) |
| Kansas | 153 (86–239) | Arkansas | 152 (88–252) |
| Georgia | 150 (87–243) | West Virginia | 151 (89–247) |
| West Virginia | 149 (85–247) | Oklahoma | 145 (86–233) |
| Maryland | 148 (86–242) | Ohio | 144 (81–237) |
| Arkansas | 147 (82–236) | South Carolina | 141 (82–242) |

DALY, disability-adjusted life year; UI, uncertainty interval; US, United States.
