# Supplementary material for: Burden of anemia in the United States from 1990 to 2019: a systematic analysis of the Global Burden of Disease Study 2019
Source: Front Public Health. 2025 Oct 3;13:1653222. doi: 10.3389/fpubh.2025.1653222 (PMC12532043; doi:10.3389/fpubh.2025.1653222)
Supplement: Supplementary file 4 [file Table_4.DOCX]

**Supplementary Table 4. Change in age-standardized DALY rates per 100,000 due to anemia in both sexes in 1990 and 2019, by state, in descending order of DALY rates in 2019.**

| **State** | **1990 DALY rates (UI)** | **2019 DALY rates (UI)** | **Change from 1990 to 2019** |
| --- | --- | --- | --- |
| Mississippi | 170 (97–277) | 175 (102–282) | + |
| District of Columbia | 260 (158–408) | 166 (98–268) | - |
| Alabama | 157 (90–254) | 158 (92–252) | + |
| Kansas | 153 (86–239) | 157 (91–258) | + |
| Louisiana | 162 (94–269) | 155 (89–250) | - |
| Arkansas | 147 (82–236) | 152 (88–252) | + |
| West Virginia | 149 (85–247) | 151 (89–247) | + |
| Oklahoma | 128 (71–211) | 145 (86–233) | + |
| Ohio | 142 (82–231) | 144 (81–237) | + |
| South Carolina | 159 (89–259) | 141 (82–242) | - |
| Georgia | 150 (87–243) | 138 (79–227) | - |
| Tennessee | 140 (82–228) | 137 (79–226) | - |
| Kentucky | 131 (74–225) | 137 (79–230) | + |
| Pennsylvania | 141 (82–225) | 135 (80–226) | - |
| Maryland | 148 (86–242) | 134 (78–220) | - |
| Delaware | 146 (83–239) | 133 (76–217) | - |
| New Mexico | 142 (81–230) | 133 (75–219) | - |
| North Carolina | 141 (80–229) | 130 (74–212) | - |
| Michigan | 132 (76–219) | 128 (73–210) | - |
| Indiana | 123 (71–200) | 127 (73–207) | + |
| Missouri | 125 (69–207) | 125 (72–205) | ~ |
| Nevada | 132 (77–214) | 124 (72–203) | - |
| Virginia | 130 (72–210) | 119 (68–198) | - |
| South Dakota | 114 (65–193) | 119 (67–202) | + |
| Florida | 120 (70–198) | 118 (69–195) | - |
| Wyoming | 118 (68–190) | 115 (67–192) | - |
| Montana | 115 (65–190) | 115 (66–188) | ~ |
| New Jersey | 130 (74–215) | 113 (66–188) | - |
| New York | 136 (80–216) | 112 (64–186) | - |
| Idaho | 114 (65–187) | 112 (63–193) | - |
| Illinois | 128 (75–212) | 112 (63–182) | - |
| Maine | 109 (61–176) | 112 (65–185) | + |
| Arizona | 117 (64–191) | 112 (63–185) | - |
| Utah | 123 (71–201) | 111 (62–187) | - |
| Massachusetts | 118 (67–191) | 111 (63–185) | - |
| North Dakota | 106 (59–172) | 110 (62–185) | + |
| Alaska | 128 (74–205) | 110 (62–182) | - |
| Rhode Island | 119 (68–195) | 110 (61–181) | - |
| Nebraska | 107 (64–185) | 108 (61–187) | + |
| Iowa | 106 (58–174) | 108 (63–180) | + |
| Texas | 111 (64–179) | 106 (60–175) | - |
| Connecticut | 113 (63–186) | 105 (60–172) | - |
| Wisconsin | 103 (59–171) | 104 (59–168) | + |
| New Hampshire | 108 (60–178) | 103 (58–178) | - |
| Vermont | 124 (72–206) | 102 (57–174) | - |
| Colorado | 109 (61–188) | 101 (58–173) | - |
| Hawaii | 109 (61–174) | 100 (57–164) | - |
| Oregon | 103 (56–170) | 99 (56–164) | - |
| Washington | 102 (56–166) | 95 (55–162) | - |
| California | 116 (66–187) | 95 (54–160) | - |
| Minnesota | 89 (49–147) | 91 (50–158) | + |

+, increase from 1990 to 2019; -, decrease from 1990 to 2019; ~ no change in estimate from 1990 to 2019, UIs may or may not have changed; DALY, disability-adjusted life year; UI, uncertainty interval.
