# Supplementary material for: Burden of anemia in the United States from 1990 to 2019: a systematic analysis of the Global Burden of Disease Study 2019
Source: Front Public Health. 2025 Oct 3;13:1653222. doi: 10.3389/fpubh.2025.1653222 (PMC12532043; doi:10.3389/fpubh.2025.1653222)
Supplement: Supplementary file 5 [file Table_5.DOCX]

**Supplementary Table 5. Recommendations and supportive data for the various causes of anemia.**

| **Anemia cause** | **Recommendation** | **Supportive data** |
| --- | --- | --- |
| Dietary iron deficiency | WHO: food-based strategies as well as food fortification to improve dietary iron intake^21^  British Society of Gastroenterology: initial treatment of IDA with oral iron, with consideration of parenteral iron treatment if oral iron is not well tolerated, ineffective, or contraindicated^32^ | Several trials showed improvements in IDA with dietary iron interventions^33^ |
| Chronic kidney disease | Kidney Disease Improving Global Outcomes: oral or IV iron based on the severity of the iron deficiency and the response and side effects with prior oral iron use^34^ | Patients with CKD and anemia treated with IV iron were more likely to achieve a hemoglobin response >1 g/dL compared with those treated with oral iron supplementation, with similar rates of adverse events and mortality^35^ |
| Gynecological diseases | WHO: iron supplementation for nonpregnant menstruating females^21^ | Females who received iron were less likely to be anemic and had higher hemoglobin concentrations compared with those who did not; iron supplementation also reduced fatigue^36^ |
| Maternal disorders | WHO: daily oral iron and folic acid supplementation for pregnant females, with intermittent supplementation in those with side effects^21^ | Iron supplementation in pregnant females halved the risk of anemia and significantly reduced the risk of iron deficiency and IDA in the third trimester or at delivery and reduced the risk of low birth weight^37^  Pregnant females with anemia successfully treated with iron showed significantly lower odds of preterm birth and preeclampsia compared with females with refractory or untreated anemia in a population-based cohort study^38^ |

CKD, chronic kidney disease; IDA, iron-deficiency anemia; IV, intravenous; WHO, World health Organization.
